# Aberrant Splicing Is the Pathogenicity Mechanism of the p.Glu314Lys Variant in *CYP11A1* Gene

**DOI:** 10.3389/fendo.2018.00491

**Published:** 2018-09-05

**Authors:** Claire Goursaud, Delphine Mallet, Alexandre Janin, Rita Menassa, Véronique Tardy-Guidollet, Gianni Russo, Anne Lienhardt-Roussie, Claudine Lecointre, Ingrid Plotton, Yves Morel, Florence Roucher-Boulez

**Affiliations:** ^1^Laboratoire de Biochimie et Biologie Moléculaire Grand Est, UM Pathologies Endocriniennes Rénales Musculaires et Mucoviscidose, Groupement Hospitalier Est, Hospices Civils de Lyon, Bron, France; ^2^Centre de Référence du Développement Génital: du Fœtus à l'Adulte, Filière Maladies Rares Endocriniennes, Bron, France; ^3^Univ Lyon, Université Claude Bernard Lyon 1, Lyon, France; ^4^Laboratoire de Biochimie et Biologie Moléculaire Grand Est, UM Cardiogénétique Moléculaire, Groupement Hospitalier Est, Hospices Civils de Lyon, Bron, France; ^5^Institut NeuroMyoGène, CNRS UMR 5310 – INSERM U1217, Université de Lyon 1, Lyon, France; ^6^Centro di Endocrinologia dell'infanzia e dell'adolescenza, Ospedale San Raffaele, Milan, Italy; ^7^Service de Pédiatrie Médicale, Hôpital de la mère et de l'enfant, CHU de Limoges, Limoges, France; ^8^Service de Pédiatrie Médicale, CHU Charles Nicolle, Rouen, France

**Keywords:** CYP11A1, alternative splicing, adrenal insufficiency, disorders of sex development, congenital lipoid adrenal hyperplasia

## Abstract

**Context:** The cholesterol side chain cleavage enzyme (CYP11A1) catalyzes the conversion of cholesterol to pregnenolone, the first rate-limiting step of steroidogenesis. *CYP11A1* mutations are associated with primary adrenal insufficiency (PAI) as well as disorders of sex development (DSD) in 46,XY patients.

**Objective:** To define the pathogenicity mechanism for the p.Glu314Lys variant, previously reported, and found in four additional patients with CYP11A1 deficiency.

**Subjects and Methods:** DNA of four patients presenting with delayed PAI and/or 46,XY DSD were studied by Sanger or Massively Parallel sequencing. Three *CYP11A1* mutations were characterized *in vitro* and *in silico*, and one by mRNA analysis on testicular tissue.

**Results:** All patients were compound heterozygous for the previously described p.Glu314Lys variant. *In silico* studies predicted this mutation as benign with no effect on splicing but mRNA analysis found that it led to incomplete exon 5 skipping. This mechanism was confirmed by minigene experiment. The protein carrying this mutation without exon skipping should conserve almost normal activity, according to *in vitro* studies. Two other mutations found in *trans*, the p.Arg120Gln and p.Arg465Trp, had similar activity compared to negative control, consistent with the *in silico* studies.

**Conclusions:** We provide biological proof that the p. Glu314Lys variant is pathogenic due to its impact on splicing and seems responsible for the moderate phenotype of the four patients reported herein. The present study highlights the importance of considering the potential effect of a missense variant on splicing when it is not predicted to be disease causing.

## Introduction

Steroidogenesis is initiated by two proteins, both expressed in adrenal and gonadal cells. First, the steroidogenic acute regulatory protein (StAR; encoded by the *STAR* gene) allows rapid cholesterol import into the inner mitochondrial membrane (IMM). Then, the cholesterol side chain cleavage enzyme (P450scc) also called CYP11A1 (encoded by the *CYP11A1* gene), converts, in the IMM, cholesterol to pregnenolone, the precursor for all steroid hormones. CYP11A1 catalyzes three reactions: 22-hydroxylation of cholesterol, 20-hydroxylation of 22R-hydroxycholesterol, and scission of the C20-22 carbon bond of 20,22R-dihydroxycholesterol. These reactions are only possible in cooperation with two cofactors, an electron transfer chain (ferrodoxin reductase) and an iron-sulfur protein (ferrodoxin) which provide electrons from NADPH (1).

Deficiency in one of these two proteins disrupts synthesis of all adrenal and gonadal steroids. It results in primary adrenal insufficiency (PAI) with various symptoms such as hypoglycemia, dehydration, weight loss or vomiting, as well as disorders of sex development (DSD) in 46,XY patients (2). The two defects are often grouped under the term of “Congenital Lipoid Adrenal Hyperplasia” (CLAH) (2). However, it is controversial since enlarged adrenal glands were not reported in CYP11A1 deficiency (1). Initially only mutations in *STAR* gene were found in patients presenting with CLAH (3, 4). As a defect in CYP11A1 does not allow progesterone synthesis during fetal life, leading to absence of uterine contraction inhibition (5), it was first considered incompatible with the maintenance of pregnancy and life (6). However, since 2001 (7), *CYP11A1* gene mutations have been found in patients presenting with a similar phenotype that is characterized by PAI with a various degree of DSD in 46,XY patients, ranging from normal female to normal male external genitalia. This defect is an extremely rare genetic autosomal recessive disorder; at the time of writing, 25 mutations of *CYP11A1* gene have been described in 29 families (corresponding to 37 patients) (7–21). Several families were reported with mild forms including delayed PAI and normal male external genitalia and were almost all homozygous for the same mutation p.Arg451Trp (14, 19). This milder phenotype could be explained by a high residual activity compared to wild-type (WT) according to functional studies (14).

Among the patients for whom we identified CYP11A1 deficiency, four were compound heterozygous for the previously described p.Glu314Lys (c.940G>A) variation (17, 21) in *trans* with two other mutations: p.Arg120Gln (c.359G>A) (17) and a new one p.Arg465Trp (c.1393C>T). The p.Glu314Lys mutation has been reported twice in a cohort of patients with PAI without clinical details (17) and also *in trans* with a splicing mutation in a patient presenting with PAI and hypospadias (21). The authors concluded to its pathogenicity on *in silico* studies showing impairment of protein secondary structure by the amino acid (AA) change and on variant segregation with the disease (17, 21). The objective of the present study was thus to characterize pathogenic mechanism of this variant. *In vitro* studies, predicted that the AA change was likely benign, however mRNA analysis found for the first time that the pathogenic mechanism of the c.940G>A (p.Glu314Lys) mutation is an aberrant splicing. The two other mutations seemed severe based on functional studies.

## Subjects and methods

### Case reports

Patients 1 and 2 were siblings from an Italian non-consanguineous family. Patient 1 was born at term with female phenotype. Ultrasonography failed to reveal uterus at 1 month, but two male gonads were found in the inguinal region. The karyotype was 46,XY. At the age of three years, ACTH level was highly elevated (473 pg/mL) and steroid levels were low and did not increase after ACTH stimulation test. Adrenal glands ultrasound was normal. Her older brother, patient 2, presented with perineal hypospadias at birth, corrected by surgery. At the time of CYP11A1 deficiency diagnosis, he had never had adrenal insufficiency.

Patient 3, the child of a non-consanguineous French family was born at term. At the age of 4 years she was admitted to hospital with abdominal pains, vomiting, asthenia, and melanoderma, suggestive of PAI. The diagnosis was confirmed by elevated ACTH (7,120 pg/mL) and very low cortisol levels (8 nmol/L). Hydrocortisone and fludrocortisone replacement was initiated. Ultrasound at the age of 9 years found a physiological pre-pubescent uterus of small size and normal sized adrenal glands. She developed attention deficit disorder but cerebral MRI was normal.

Patient 4 was born at term without fetal distress, from a non-consanguineous French family. After many episodes of shock, at 3 years and 8 months of age she presented with melanoderma, recurrent abdominal pains, hypoglycemia, and hyponatremia (123 mmol/L). Diagnosis of PAI was made with elevated ACTH (>1,900 pg/mL), and low 17-hydroxyprogesterone and cortisol levels. Although aldosterone was normal, initial glucocorticoid- and mineralocorticoid-replacement was maintained because of slightly increased renin activity. Each suspension of mineralocorticoid replacement was associated with the increase of renin. Karyotype was 46,XX. The adrenal CT scan performed at the age of 6 years found neither calcification nor morphological abnormality. At 8 years of age, an onset of puberty (S2) occurred which was treated by GnRH analog for 2 years. Menarche occurred at 12 years of age and was followed by regular menstrual cycles. At 16 years of age, she presented with secondary amenorrhea due to anorexia nervosa until the age of 23 years. At 25 years of age, she had no contraception, regular menstrual cycles and normal hormonal data at the beginning of her cycle (LH: 3IU/L; FSH: 6IU/L). Pelvic ultrasonography showed normal sized ovaries with several follicles (*n* = 11 and 14).

### DNA sequencing

The study was conducted in accordance with the principles of the Declaration of Helsinki and was approved by the Local Ethics Committee of the Hospices Civils de Lyon. Written informed consent was provided by all parents of the patients enrolled in the study. Genomic DNA was extracted from EDTA-preserved whole blood using Nucleon BACC3 kit (GE healthcare, Chalfont Saint Giles, Buckinghamshire, UK). DNA was analyzed by Sanger sequencing or Massively Parallel sequencing (MPS).

For patients 1 and 2, as CLAH was suspected on clinical and biological data, *STAR* and *CYP11A1* genes were immediately Sanger sequenced. For patients 3 and 4 who presented with glucocorticoid deficiency, *MC2R* and *MRAP* genes were first Sanger sequenced. DNA was further analyzed by MPS.

Sanger sequencing consisted of selective amplification of the exons and the exon-intron boundaries of the analyzed gene by PCR using specific primers (sequences available upon request), followed by conventional dideoxy sequencing on an ABI-3500XL sequencer (Thermofisher scientific, Watham, MA, USA) and compared to the human genome (GRCh37/hg19 assembly) using SeqScape® software v3 (Thermofisher scientific).

For MPS, a custom panel targeting 57 genes involved in adrenal insufficiency and DSD including the *CYP11A1* gene as previously described (22) was used. Pathogenic mutations found by MPS were verified by Sanger sequencing.

### Pathogenicity prediction

#### Alamut software

Pathogenicity prediction was performed *in silico* using Alamut software v2.9-0 (Interactive Biosoftware, Rouen, France), which includes several pathogenicity prediction programs: align GVGD, Polyphen 2, Mutation Taster, SIFT, splice site prediction methods and ESE binding site detection. The Grantham score that ranges from 0 to 215, was calculated to predict the effect of substitutions between AA based on chemical properties (i.e., polarity and molecular volume). Higher scores indicate greater differences between two AA and may indicate a stronger (negative) effect on protein structure and function. Frequency databases (dbSNP, ESP, and gnomAD) were searched.

#### Multiple sequence alignment

Multiple sequence alignments were performed to analyze structurally conserved regions and to predict putative effects of missense mutation: human CYP11A1 alignment with orthologs or with other human cytochromes involved in steroidogenesis. Sequences were found in the Uniprot database (http://www.uniprot.org/), aligned with Clustal Omega (https://www.ebi.ac.uk/Tools/msa/clustalo/) using default parameters, displayed and then edited using Genedoc (Free Software Foundation, Inc., Boston, MA, USA).

#### Three-dimensional molecular modeling

The crystal structure of human CYP11A1 in complex with cholesterol (https://www.rcsb.org/pdb, PDB code 3N9Y) was used to identify secondary structure sequences, and AA involved in important structures—such as membrane interaction domain, heme binding domain (including cysteine pocket), active site, substrate recognition site (SRS), and redox partner interaction domain—and analyze the impact of mutations on the three-dimensional structure using DeepView—the PDB Viewer (GlaxoSmithKline Research and Development, S.A., Geneva, Switzerland).

### Construction of CYP11A1 mutant plasmid

The three mutants p.Glu314Lys, p.Arg465Trp, and p.Arg120Gln and a known nonsense mutant p.Arg120Stop (used as negative control) (16) were created in the F2 plasmid (kindly provided by Prof. W. L. Miller, Department of Pediatrics, University of California, San Francisco, CA, USA) expressing the fusion protein NH2-CYP11A1-Ferrodoxin-reductase-Ferrodoxin-COOH (23). Mutations were introduced by site directed mutagenesis using “Quick Change II XL Site-directed mutagenesis” (Agilent Technologies, Santa Clara, CA, USA). The methylated parental (WT) cDNA was digested with Dpn I at 37°C for 1 h and transformed into ultra-competent Escherichia coli XL10-Gold. Mutations insertion and cDNA integrity were verified by sequencing.

### Functional CYP11A1 activity assay

COS 1 cells seeded in six-well plate were transfected with either the WT, the mutant F2 plasmid or an empty vector using FugeneHD transfection reagent according to the manufacturer's protocol (Promega, Madison, WI, USA) at ~80% confluence. After 48 h of incubation with transfection reagent in DMEM containing 10% fetal calf serum and antibiotics, cells were incubated at 37°C for 24 h with 22R-hydroxycholesterol, used as substrate because it does not require StAR to enter the mitochondria, where it is converted to pregnenolone by P450scc. Several concentrations of substrate were used to determine kinetic constants of each mutant: 0.5, 1, 1.5, 2, 3, and 5 μmol/L. Pregnenolone was quantified by high performance liquid chromatography/tandem mass spectrometry.

For total protein measurements, cells were detached from plates by incubation with trypsin-EDTA for 10 min. Trypsin action was then stopped by addition of complete culture medium. After centrifugation, the cell pellet was washed with PBS and proteins were extracted using complete Lysis-M reagent (Roche diagnostics, Mannheim, Germany) and measured by a bicinchoninic acid (BCA) assay using an ABX Pentra 400 analyzer (Horiba Scientific, Kyoto, Japan).

The CYP11A1 activity of each mutant was defined as a percentage of substrate conversion, after normalization to total protein measurements, compared to the WT activity that was defined as 100%. Enzymatic activities of each mutant were compared with WT and negative controls (the p.Arg120Stop mutant or the empty vector) at a substrate concentration of 3 μmol/L incubated for 24 h, using Anova 1 test followed by Tukey's multiple comparison test. Michaelis–Menten analyses were performed using graphpad prism software v5.0 (GraphPad, Inc., San Diego, CA, USA).

### mRNA analysis

Patient 1 had gonadectomy after diagnosis. Total RNA was isolated from testicular tissue using the RNA Now kit (Biogentex Laboratories, Inc., League City, TX, USA), reverse transcribed to cDNA with random hexamers and amplified using Gene Amp RNA PCR kit (Thermofisher scientific). Several primers (designed from sequences of exons 3, 6, and 9, intron 6, and overlapping exons 4 and 6) were used for PCR and sequencing (primers sequences available on request).

### Minigene splicing reporter assay

*CYP11A1* exon 5 with 313 flanking intronic bases in intron 4 and 241 flanking intronic bases in intron 5 was PCR amplified from the patient genomic DNA (total product size: 714 bp). Wild-type (WT) and mutated polymerase chain reaction (PCR) products were inserted in the *Nde*I restriction site of the pTB minigene vector as previously reported (24, 25). Transfection in HeLa cells, reverse transcription -PCR (RT-PCR) procedures and analysis have been previously described (24, 25). Reverse transcription-PCR products were analyzed by agarose gel electrophoresis (2%) and Sanger sequencing.

## Results

### DNA sequencing (Figure 1)

As no disease-causing mutations were found in *STAR* (patients 1 and 2), *MC2R or MRAP* genes (patients 3 and 4), *CYP11A1* gene was sequenced using the Sanger method or MPS. The p.Glu314Lys (c.940G>A) variant was found in all patients at heterozygous state. Patients 1, 2, and 3 carried in *trans* the p.Arg465Trp (c.1393C>T) variant (haplotype segregation was confirmed by parental analysis) and patient 4 was supposed to be compound heterozygous for p.Glu314Lys and p.Arg120Gln (c.359G>A) mutations. The missense variant p.Glu314Lys in exon 5, as the variant p.Arg120Gln in exon 2, were previously reported without functional analysis (17, 21). The p.Arg465Trp mutation in exon 8 has never been reported.

**Figure 1:**
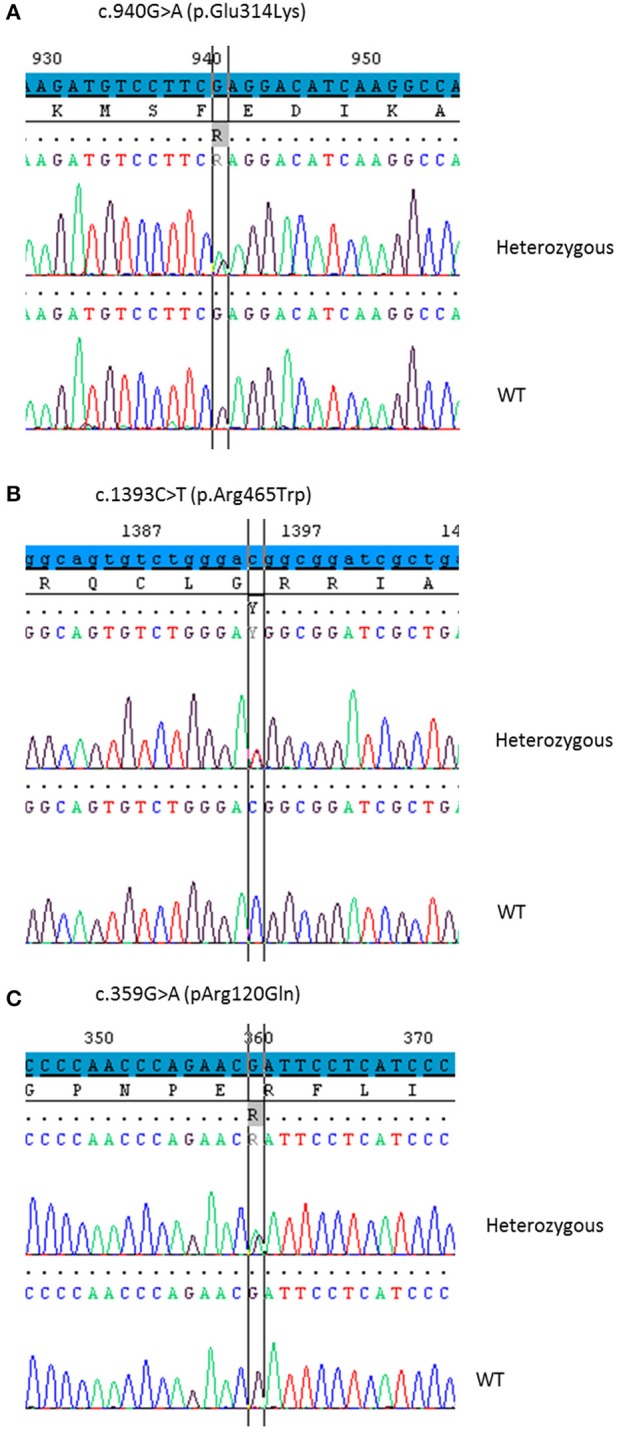
Partial chromatograms showing the *CYP11A1* mutations in heterozygous state detected in the four patients. DNA sequencing of a healthy control shows the WT sequence underneath. **(A)** The base change c.940G>A leads to the missense mutation p.Glu314Lys. **(B)** The base change c.1393C>T leads to the missense mutation p.Arg465Trp. **(C)** The base change c.359G>A leads to the missense mutation p.Arg120Gln.

### Pathogenicity characterization (Table 1)

**Figure 2:**
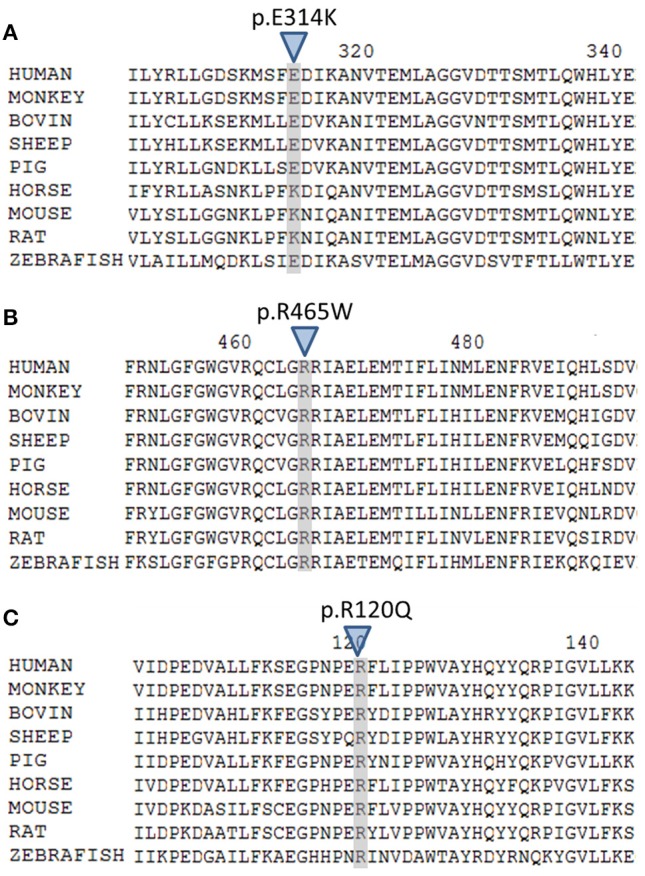
Multiple alignment of human CYP11A1 with orthologs. **(A)** The E314 residue shaded and marked by a triangle is slightly conserved across species. **(B)** The R465 residue shaded and marked by a triangle is highly conserved across species. **(C)** The R120 residue shaded and marked by a triangle is highly conserved across species.

**Figure 3:**
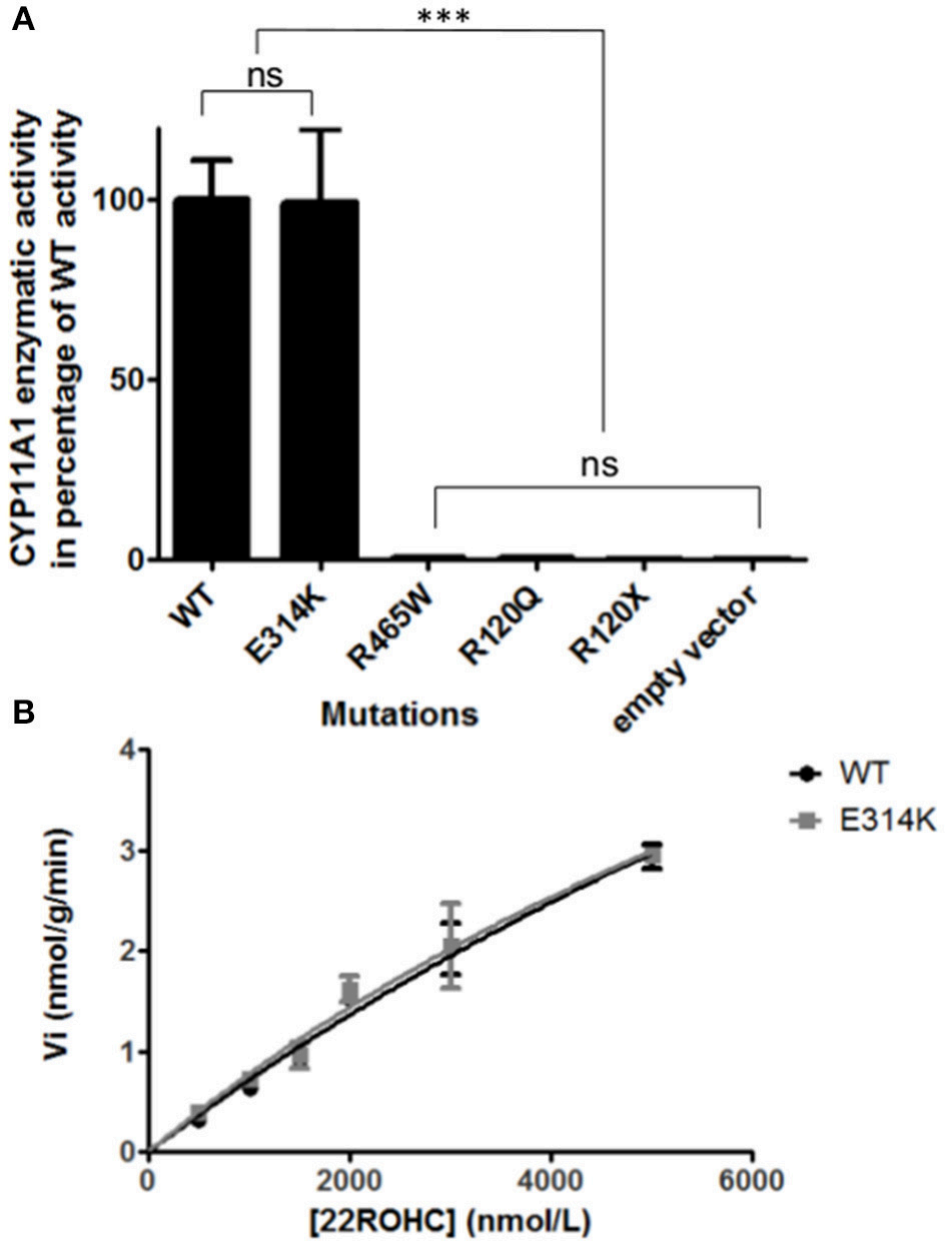
*In vitro* functional CYP11A1 activity assays. **(A)** Comparison of mutant's residual activity to WT and negative control's activity. Enzymatic activities are reported as % compared to WT activity. Assays were performed after incubation of transfected cells with 3 μmol/L of 22R-hydroxycholesterol in three independant triplicate experiments. Pregnenolone was measured by HPLC-MS/MS. The data are shown as mean ± s.e.m. Enzymatic activities were compared using Anova 1 and Tukey's multiple comparison tests on Graphpad prism software v5.0 (GraphPad, Inc). No significant difference in activity was found between p.Glu314Lys and WT (*p* ≥ 0.05). The p.Arg120Gln and p.Arg465Trp mutants had residual activity similar to negative controls (p.Arg120Stop and empty vector) (*p* ≥ 0.05). ns, not significantly different (*p* ≥ 0.05). ^***^Significantly different (*p* < 0.0001). **(B)** Michaelis Menten representation of p.Glu314Lys (in gray) and WT's (in black) activity. Assays were performed after incubation of transfected cells with 0.5, 1, 1.5, 2, 3, 5μmol/L of 22R-hydroxycholesterol in three independant triplicate experiments. Pregnenolone was measured by HPLC-MS/MS. The data are shown as mean ± s.e.m.

The variant p.Glu314Lys (c.940G>A) was predicted likely benign by three of the four prediction software. Moreover, the residue at this position is slightly conserved between species (Figure 2A) and the Grantham score of the mutation was low although the residues differ in polarity, charge, and size. This variant, reported in dbSNP (rs6161), has a minor allele frequency (MAF) of 0.34% in ESP and 0.26% in gnomAD (0.42% in the European population). Splicing predictor software did not find acceptor and donor site score modification or cryptic splice site creation. The 3D modeling found that residue Glu314 is located at the beginning of the I helix in the center of which is located the active site, a SRS, and a heme binding domain. There seemed to be no impact of residue modification on any of these domains. This is consistent with *in vitro* studies as no significant difference in activity was found between p.Glu314Lys and WT (*p* ≥ 0.05; Figures 3A, B). However, this variant was previously reported twice in the literature in three unrelated patients who had a phenotype concordant with *CYP11A1* deficiency (17, 21). It was associated in *trans* with pathogenic mutation for two out of the three patients. Therefore, according to the standards and guidelines of the American College of Medical Genetics and Genomics (26) this variant should be classified as probably pathogenic.

**Table 1:** Pathogenicity prediction and enzymatic activity values of the three *CYP11A1* mutations.

**Nucleotide change**	**Exon**	**Protein change**	**Protein consequence (3D model)**	**Domain**	**Grantham score [0–215]**	**Prediction software**				**db SNP ID**	**Allele count**		**Enzymatic activities (%/WT)**	**References**
						**GVGD**	**SIFT**	**Polyphen-2**	**Mutation Taster**		**ESP**	**gnomAD**		
c.940G>A	5	p.Glu314Lys	Unknown	I helix	56	Less likely pathogenic C0	Tolerated	Benign	Disease causing	rs6161	37/12949	710/277190	99.20%	(17, 21)
c.1393C>T	8	p.Arg465Trp	Disturbance of electrostatic bounding with redox partner	L helix	101	Less likely pathogenic C0	Deleterious	Probably damaging	Disease causing	rs141235847	1/12989	6/246116	0.60%^*^	
c.359G>A	2	p.Arg120Gln	Loss of H-bond involved in heme binding	B-B′ loop	43	Intermediate C35	Deleterious	Probably damaging	Disease causing	Unknown	Unknown	Unknown	0.40%^*^	(17)

Enzymatic activities are reported as % compared to WT activity.

* *Not significantly different from negative control (p ≥ 0.05). Enzymatic activities were compared using Anova 1 and Tukey's multiple comparison tests on graphpad prism software v5.0 (GraphPad, Inc)*.

The new p.Arg465Trp (c.1393C>T) variant is predicted probably damaging by three out of the four software. It is reported with a very low frequency in ESP (0.01%) and gnomAD (All: 0.0024%, European: 0.0045%) databases. Physicochemical effect of this variation is important with a Grantham score of 101. Residue Arg465 is located at the beginning of the L helix near the cysteine pocket, which allows covalent bonding with the heme. Amino acid Arg465 is involved in redox partner interaction and is highly conserved between species (Figure 2B). The replacement of a basic AA by a neutral residue should disturb electrostatic bounding with the redox partner. In addition, among the patients with CYP11A1 deficiency that we had identified, one was homozygous for this mutation and had a severe phenotype (early onset of PAI and severe DSD). Functional studies found a residual activity like negative control (*p* ≥ 0.05; Figure 3A), which is in accordance with *in silico* prediction (kinetic studies were not performed owing to this very weak activity). Therefore, this variant should be pathogenic.

**Figure 4:**
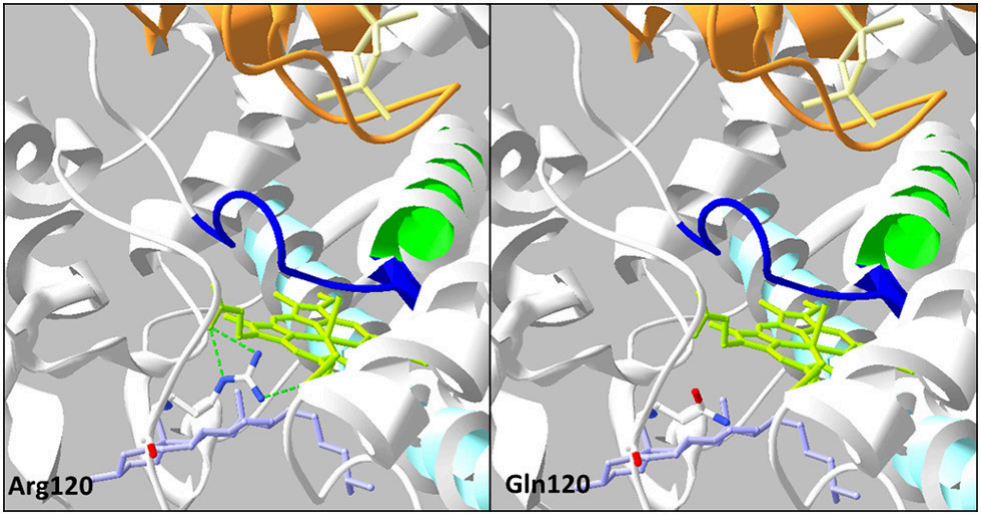
Analysis of the mutation p.Arg120Gln on three-dimensional model of CYP11A1 (PDB: 3N9Y). This amino acid replacement leads to disruption of three H-bonds (green dotted line) with the heme in green anis. The cholesterol is in violet, the I helix in light blue, L helix in green, cystein pocket in deep blue, and ferrodoxin in orange.

The p.Arg120Gln (c.359G>A) variant is predicted deleterious by the four pathogenicity prediction software. This variation was not listed in any databases. Residue Arg120 is located in the B-B' loop, is highly conserved between species (Figure 2C), and is in the heme binding domain and the SRS; molecular modeling predicted that the mutation disrupts three H-bonds made with the heme (Figure 4). This mutation was previously reported in a patient presenting with familial glucocorticoid deficiency (17). Functional studies found a residual activity like negative control (*p* ≥ 0.05; Figure 3A) which is in accordance with *in silico* prediction (kinetic studies were not performed owing to this very weak activity). Therefore, this variant seems to be pathogenic.

### mRNA analysis of patient 1

**Figure 5:**
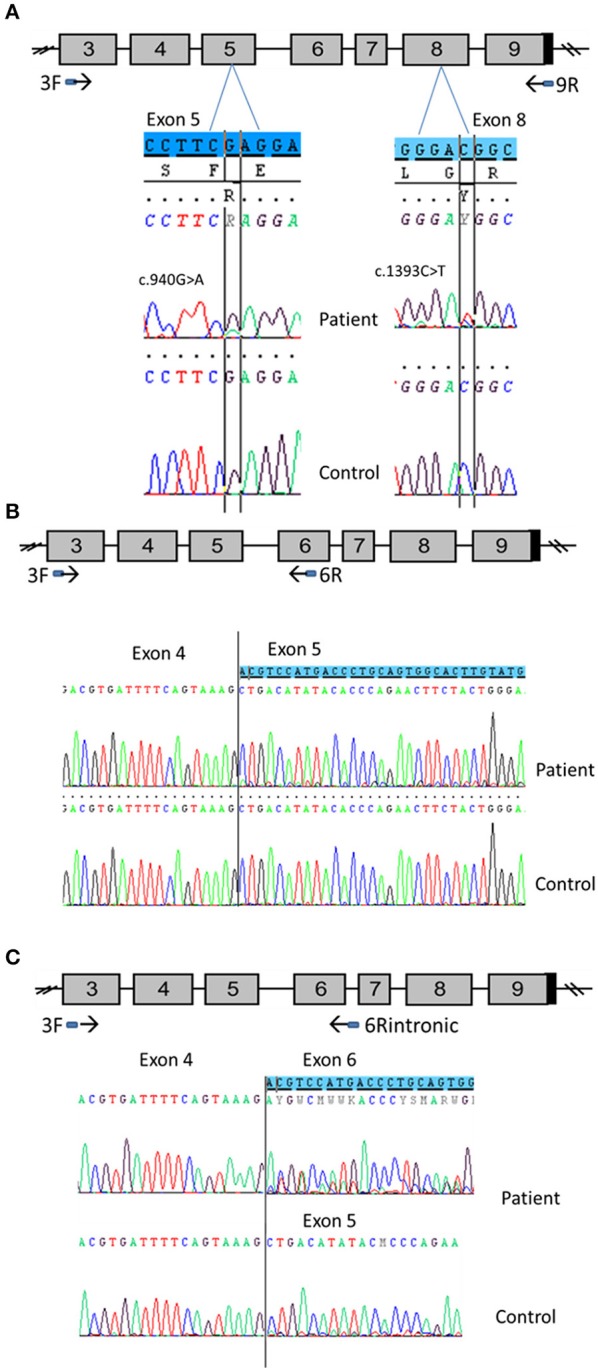
CYP11A1 mRNA analysis from testicular tissue of patient 1 compound heterozygous for the p.Glu314Lys and p.Arg465Trp mutations. **(A)** Amplification of CYP11A1 exons 3–9 (the reference sequences of exon 5 and 8 are highlighted in blue). Sequences of patient cDNA found the c.940G>A mutation in exon 5 and the c.1393C>T mutation in exon 8 at heterozygous state. WT nucleotide is more important than mutated nucleotide in exon 5 and in contrast, there was more mutated than WT nucleotide in exon 8. **(B)** Amplification of CYP11A1 exons 3–6. Sequence of patient exon 5 found a low-level second sequence, which matches sequence of exon 6 (the reference sequence of exon 6 is highlighted in blue). This is not the case for the control sample (underneath). **(C)** Amplification of exon 3 to intron 6. Sequence of patient cDNA is above and shows the skipping of exon 5 at heterozygous state (the reference sequence of exon 6 is highlighted in blue). Sequence of WT, underneath, shows this skipping at a very low level.

To evaluate the discrepancy between *in vitro* functional studies that found normal activity for the p.Glu314Lys variant, and the relationship with CYP11A1 deficiency, mRNA analysis from available testicular tissue of patient 1 was performed (Figure 5).

First exons 3–9 were amplified. Sequencing of the PCR product found the mutation c.940G>A in exon 5 (responsible for Glu314Lys) at heterozygous state. However, there was more WT than the mutated nucleotide (Figure 5A). This was not mosaicism, because sequencing of patient gDNA found nearly equal WT and mutated nucleotides (Figure 1). It also found the mutation c.1393 C>T in exon 8 (responsible of Arg465Trp change at protein level). In contrast, there was more mutated than WT nucleotide (Figure 5A).

Taken together, these two results are in favor of an incomplete degradation of allele carrying the c.940G>A mutation. It may be degraded by Nonsense-Mediated mRNA Decay (NMD) due to abnormal splicing. Therefore, primers which were designed from sequences of exons 3 and 6, surrounding exon 5, where the mutation is located, were used to amplify cDNA to better appreciate the effect of the mutation and visualize low-level aberrant splicing. For the patient cDNA, two fragments were obtained, the smallest one was more intense, whereas only one fragment was found for the control sample. Again, sequencing of PCR products found more WT than the mutated nucleotide for the patient. Above all, it found a low-level second sequence beginning in exon 5 that matched with exon 6 reference sequence which was not detected in control sample (Figure 5B). These results confirmed the exon 5 skipping, leading to a frameshift and creation of a premature stop codon (r.830_990del, p.Ala277Aspfs^*^11).The low level of the second sequence (corresponding to exon 5 skipping) and the lower level of mutated nucleotide should be explained by NMD action.

Investigation of the splicing sites of the *CYP11A*1 gene found that the donor sequence of intron 6 has nucleotides “GC” instead of usual “GT” donor site. Moreover, this donor site is recognized by only one splicing predictor software, which is in favor of a weak donor site. Assuming intron 6 retention, a fragment of cDNA was amplified using primers which were designed from sequences of exon 3 and intron 6. This amplification found two fragments for both the patient and control samples; the longest fragment was less intense than the shorter fragment in the patient sample and the converse was found in the control sample. This amplification in patient and control using an intronic primer highlights physiologically intron 6 retention. Sequencing PCR product showed in the patient sample a second sequence aligned on exon 6 of CYP11A1 template and no sequence on exon 5 highlighting exon 5 skipping in a heterozygous state; and a similar exon skipping but at a lower level in the control sample (Figure 5C). Thus, we demonstrate that c.940G>A mutation favors exon 5 skipping. However, this exon skipping seems to exist physiologically at a low level since it is present in the control sample.

This hypothesis was verified by amplification using the same primer in exon 3 and a primer overlapping exons 4 and 6. Amplification was obtained for patient and control samples, confirming exon 5 skipping in patient but also physiologically (data not shown), probably at a very low level.

### Minigene splicing reporter assay

In order to prove the causal relationship between exon 5 skipping and the c.940G>A mutation, a minigene assay was performed: the 714 bp genomic fragment containing the variation (exon 5 with flanking intronic bases in introns 4 and 5) was inserted in the pTB minigene vector (27). In addition, the wild-type sequence was also inserted in the pTB minigene vector, used as control.

**Figure 6:**
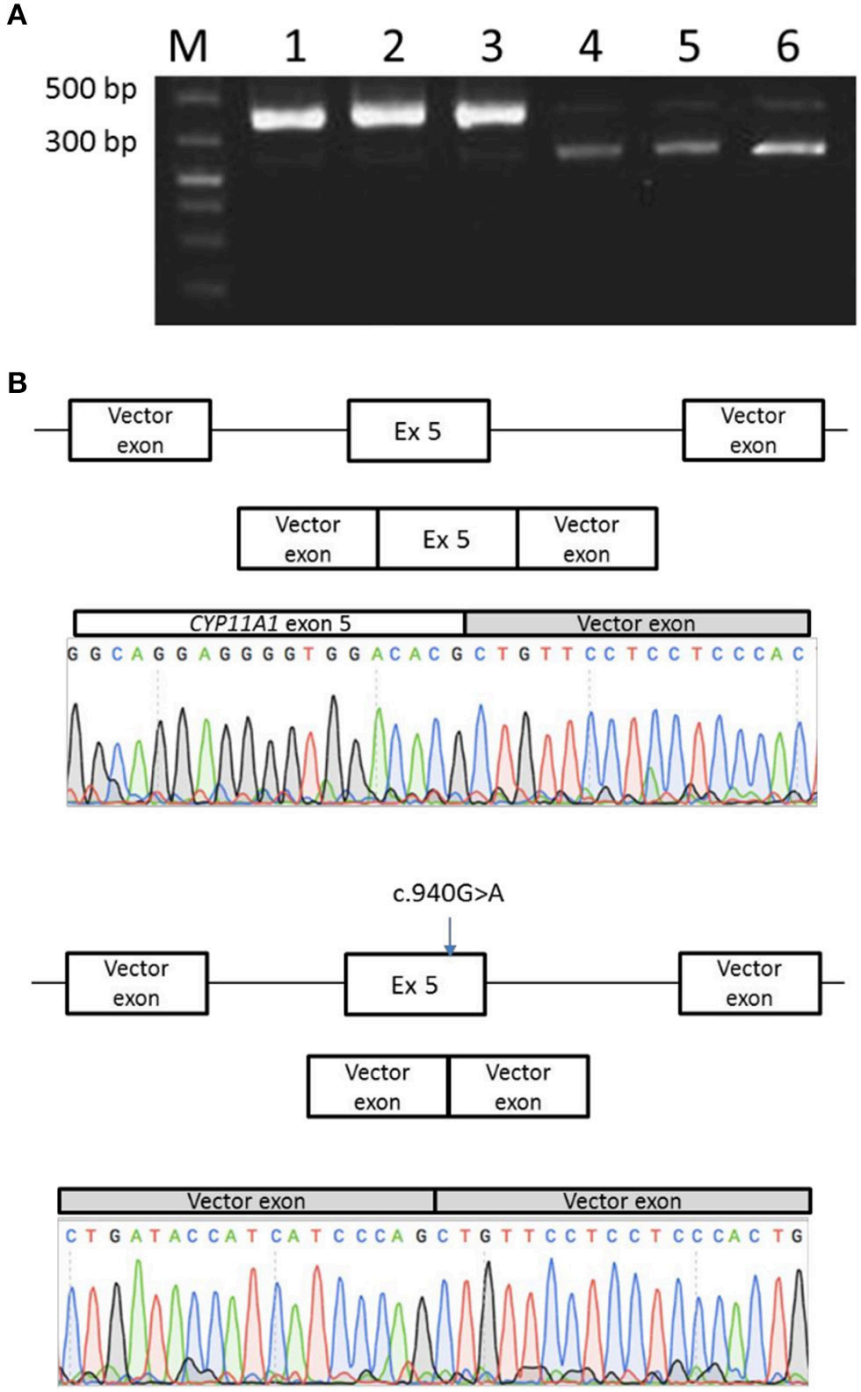
Minigene splicing assay for the p.Glu314Lys mutant. **(A)** Transfection of the mutant minigene (wells 4, 5, and 6) showed a shorter RT-PCR product in comparison with normal minigene (wells 1, 2, and 3). **(B)** Sequencing of PCR products revealed normal splice product for the normal minigene and a complete exon skipping for the mutant minigene.

Transfection of the mutant minigene showed two different RT-PCR products: a first one, similar in size to the RT-PCR product obtained with the normal minigene, and a shorter one, which is in greater quantities than the normal one. Sequencing of both RT-PCR products revealed indeed complete exon skipping for the mutant minigene and a normal spliced product for the minigene with wild-type sequence (Figure 6).

These results confirmed thus that this exon 5 skipping phenomenon is associated with pathogenicity of the c.940G>A allele.

## Discussion

CYP11A1 deficiency is a rare disorder; less than 40 patients have been reported previously and the p.Glu314Lys mutation was reported twice in three unrelated families (17, 21). To date the pathogenic role of this mutation relies mainly on segregation and allelic data (17, 21). Herein we report four additional patients carrying this variant and data from mRNA studies that confirm that aberrant splicing is the pathogenicity mechanism.

The impact on splicing should be considered when a missense mutation is predicted likely benign but which seems related to the disease in unrelated families. Indeed, the p.Glu314Lys (c.940A>G) variant was misinterpreted by the majority of prediction tools considering it as benign with no effect on splicing and a non-null MAF in databases. Moreover, *in vitro* studies went in the same way showing a similar enzymatic activity for the mutated protein than for the WT protein. The *in silico* data herein are also concordant as no impact on protein structure was found. Taken together, these data do not support the hypothesis proposed by Lara-Velazquez et al. who suggest that this variant may disrupt cholesterol binding (21). The pathogenicity was finally highlighted thanks to mRNA analysis on testis tissue which found aberrant splicing with exon 5 skipping. The exact mechanism of exon skipping remains unknown, even though we suspect modification of Exonic Sequence Enhancer (ESE) which could disturb splicing as it has already been reported for other exonic mutations (28). This exon skipping should lead to a frameshift and create a premature stop codon downstream (r.830_990del, p.Ala277Aspfs^*^11), activating NMD responsible for degrading this allele. The skipping is however incomplete as the c.940A>G mutation is still seen on cDNA, and the resultant protein should conserve almost normal activity.

The mRNA analysis revealed an alternative splicing occurring with the retention of at least the beginning of intron 6, probably protecting the transcript with exon 5 skipping from NMD. Indeed, when selecting transcript with intron 6 retention (which could be explained by the known unusual “GC” donor site) by the use of an intronic primer, exon 5 skipping was observed for both the patient and the control, but was to a greater level in the patient. The exact mechanism of exon 5 skipping transcript protection remains difficult to explain since this should still lead to a premature stop codon in exon 6 before intronic retention and thus activate the NMD system.

As exon skipping has been also found at a low level in control sample, minigene experiment was performed to prove the causal relationship between this phenomenon and the mutation. Indeed, it confirmed the aberrant splicing due to the c.940G>A mutation.

A good genotype-phenotype correlation can be established thanks to functional studies allowing future genetic counseling. Indeed, the two mutations p.Arg465Trp and p.Arg120Gln, found *in trans* were predicted to be severe by *in silico* analysis, and this was consistent with *in vitro* studies showing lack of activity. Moreover, the 46,XY patient homozygous for the p.Arg465Trp mutation has a severe typical phenotype of the disease. Therefore, the p.Glu314Lys variant should determine the phenotype for all carriers identified in our center. Indeed, the late onset of PAI for these patients correlates with the moderate p.Glu314Lys mutation. However, testosterone biosynthesis was not sufficient during fetal life as evidenced by the severe DSD of the 46,XY patients described herein, as was the case for the patient reported elsewhere (21). In contrast, the impact on ovarian function seems mild as attested by normal puberty and regular menses of patient 4.

These dissociated forms could be explained by the differential expression of *CYP11A1* gene in different tissues; the hypothesis being tissue-dependent and a greater aberrant splicing in testicular tissue for this mutation. To verify this hypothesis, and because study mRNA from adrenal tissue seems to be impossible, it could be interesting to perform minigene experiments in different types of affected cells to compare splicing effect of this mutation on gonadal and adrenal tissue.

In conclusion, the present study provides the biological proof that the mutation NM_000781.2(CYP11A1):c.940G>A is pathogenic due to its splicing impact and not the AA change (p.Glu314Lys). When a missense mutation predicted benign seems related to the disease, impact on splicing should be considered. The mRNA studies, when possible, should complete the investigation.

## Ethics statement

This study was carried out in accordance with the recommendations of the Local Ethics Commitee of the Hospices Civils de Lyon. The protocol was approved by the Local Ethics Committee of the Hospices Civils de Lyon. All subjects gave written informed consent in accordance with the Declaration of Helsinki. Patients provided written informed consent for the publication of this case report.

## Author contributions

CG, DM, RM, VT-G, IP, YM, and FR-B: study design. GR, AL-R, and CL: clinical work-up. CG and DM: mRNA analysis. CG: *in vitro* studies. AJ: minigene assay. CG, DM, RM, VT-G, IP, YM, and FR-B: data analysis and interpretation. CG, DM, YM, and FR-B: manuscript preparation. All authors: manuscript approval.

### Conflict of interest statement

The authors declare that the research was conducted in the absence of any commercial or financial relationships that could be construed as a potential conflict of interest.
